# Dairy Consumption and Inflammatory Bowel Disease among Arab Adults: A Case–Control Study and Meta-Analysis

**DOI:** 10.3390/nu16162747

**Published:** 2024-08-17

**Authors:** Anas M. Almofarreh, Haytham A. Sheerah, Ahmed Arafa, Ahmed S. Al Mubarak, Aidrous M. Ali, Nasser M. Al-Otaibi, Mohammed A. Alzahrani, Atif R. Aljubayl, Mohammad A. Aleid, Suliman S. Alhamed

**Affiliations:** 1 Assistant Deputyship for Health Investment Development, Ministry of Health, Riyadh 11451, Saudi Arabia; aalmofarreh@moh.gov.sa; 2 Office of the Vice Minister of Health, Ministry of Health, Riyadh 11451, Saudi Arabia; 3 Department of Preventive Cardiology, National Cerebral and Cardiovascular Center, Suita 564-8565, Japan; 4 Department of Public Health and Community Medicine, Faculty of Medicine, Beni-Suef University, Beni-Suef 62521, Egypt; 5 Medical Referrals Center, Ministry of Health, Riyadh 11451, Saudi Arabia; asabdelrahman@moh.gov.sa (A.S.A.M.); amagzoub@moh.gov.sa (A.M.A.); 6 Mystery Visitor Program, Education and Development Department, Ministry of Health, Riyadh 11451, Saudi Arabia; nal-otaibi@moh.gov.sa; 7 School of Medicine, Albaha University, Al Baha 11451, Saudi Arabia; 438002054@stu.bu.edu.sa; 8 Assistant Deputyship for International Collaborations, Ministry of Health, Riyadh 11451, Saudi Arabia; aralmutiri@moh.gov.sa; 9 General Administration for Health Facilities Licensing, Ministry of Health, Riyadh 11451, Saudi Arabiassalhamed@moh.gov.sa (S.S.A.)

**Keywords:** case–control study, Crohn’s disease, dairy, meta-analysis, ulcerative colitis, systematic review

## Abstract

Background: Inflammatory bowel disease (IBD), which includes ulcerative colitis (UC) and Crohn’s disease (CD), is a complex disease with increasing global incidence and prevalence. Although dairy consumption has been linked to various chronic diseases, its relationship with IBD remains uncertain. Additionally, there is a lack of data on this topic from Arab countries. This study aimed to investigate the association between dairy consumption and IBD through a case–control study among Arab populations, followed by a meta-analysis of available studies. Method: First, we used data from 158 UC patients, 244 CD patients, and 395 controls attending a polyclinic in Riyadh, Saudi Arabia. All participants were aged ≥ 18 years. Logistic regression was used to calculate the odds ratios (ORs) and 95% confidence intervals (95% CIs) of UC and CD for individuals who reported the highest versus the lowest frequencies of dairy consumption. Next, we conducted a meta-analysis, combining our results with those from other eligible studies after searching several databases. We used the *I*^2^ statistics to examine statistical heterogeneity across studies and the regression test for funnel plot asymmetry to assess publication bias. Results: The case–control study showed a negative association between frequent dairy consumption and UC (OR (95% CI) = 0.64 (0.41, 1.00)) but not CD (OR (95% CI) = 0.97 (0.65, 1.45)). In the meta-analysis, the highest frequencies of dairy consumption were negatively associated with both UC and CD: ORs (95% CIs) = 0.82 (0.68, 0.98) and 0.72 (0.59, 0.87), respectively. A moderate heterogeneity across studies was noticed in the UC meta-analysis (*I*^2^ = 59.58%) and the CD meta-analysis (*I*^2^ = 41.16%). No publication bias was detected. Conclusions: Frequent dairy consumption could protect against the development of UC and CD, suggesting potential dietary recommendations in the context of IBD prevention.

## 1. Introduction

Inflammatory bowel disease (IBD), encompassing ulcerative colitis (UC) and Crohn’s disease (CD), represents a group of chronic inflammatory conditions that affect the gastrointestinal tract [1]. These diseases are characterized by periods of active inflammation and remission, causing symptoms such as abdominal pain, diarrhea, rectal bleeding, weight loss, and fatigue [1]. The incidence and prevalence of IBD have been steadily rising globally, making it a significant public health challenge. This increase is observed not only in Western countries, where IBD has traditionally been more common, but also in newly industrialized and developing nations, suggesting a possible link to changing environmental and lifestyle factors [2,3].

The etiology of IBD is complex and multifactorial, involving a combination of genetic, environmental, and immunological factors. Genetic predisposition plays a crucial role, with numerous susceptibility genes identified that contribute to the disease’s development. Environmental factors, such as smoking, antibiotic use, and urbanization, have also been implicated. Among these, diet is considered a particularly influential environmental component. Specific dietary components, such as high-fat and high-sugar foods, have been associated with an increased risk of IBD, while dietary fiber and certain micronutrients may offer protective effects [4,5,6,7].

Among various dietary factors, dairy products, such as milk, cheese, and yogurt, a staple in many diets worldwide, have garnered significant attention. The biological mechanisms through which dairy consumption might influence IBD are multifaceted. On the one hand, lactose intolerance can exacerbate gastrointestinal symptoms, potentially triggering inflammatory processes in predisposed individuals [8]. Proteins in dairy, like casein and whey, may provoke immune responses and contribute to gut inflammation. Dairy consumption can also impact the gut microbiome, disrupting the balance of bacterial species and influencing inflammation. Additionally, saturated fats in dairy may alter gut barrier function and permeability, leading to increased bacterial translocation and further inflammation [9,10]. On the other hand, dairy products contain bioactive peptides that modulate immune responses and inhibit inflammation. They are also rich in calcium and vitamin D, which are beneficial for gut health. Calcium helps maintain gut barrier integrity, while vitamin D supports immune function and has anti-inflammatory effects. Probiotics in fermented dairy products can promote a healthy gut microbiome. These factors contribute to the potential protective effects of dairy against the development of IBD [9].

Evidence on the association between dairy consumption and IBD remains inconclusive due to conflicting results of epidemiological studies [11,12,13,14,15,16,17,18,19,20,21,22,23,24,25,26,27,28,29,30,31,32,33,34,35,36,37,38,39,40]. While some studies indicated that dairy consumption could offer a protective effect against IBD [11,12,18], other studies showed no association between dairy consumption and IBD [14,19] or even increased IBD risk alongside the increasing dairy consumption [27]. Additionally, some studies did not include a sufficient number of cases to achieve a statistically significant association [16,22]. In addition, most of these studies were conducted on Western and East Asian populations, limiting their generalizability to other populations.

In Arab countries, dairy consumption is a significant cultural practice. Dairy product sales in the Gulf Cooperation Council countries increased by 50% between 2007 and 2012, with the Saudi market accounting for 74% of this volume. Saudi consumers also demonstrated the highest per capita dairy consumption [41]. Still, the role of dairy consumption in the development of IBD among Arab populations remains understudied.

Given the absence of data on the association between dairy consumption and IBD in Arab populations, we conducted a case–control study at a Saudi polyclinic to examine this association. Due to inconsistent findings across previous studies and the insufficient number of IBD cases in some research, we also performed a systematic review of the literature on the relationship between dairy consumption and the risk of IBD. By combining our study results with those from previous studies in a meta-analysis, we aimed to draw more definitive conclusions about the role of dairy consumption in the development of UC and CD.

## 2. Methods

### 2.1. The Case–Control Study

#### 2.1.1. Study Design, Population, and Setting

Sample size calculation was conducted using Epi Info based on the following: two-sided confidence level (1-alpha): 95; power (% chance of detecting): 80; the ratio of controls to cases: 2; hypothetical proportion of controls with exposure: 50; hypothetical proportion of cases with exposure: 30; and the least extreme odds ratio (OR) to be detected: 0.43. The calculated sample size was 73 for cases and 146 for controls, given that UC and CD were investigated separately. We more than doubled the sample size to account for the potential lack of data on dairy consumption and to enable stratified results by sex. For this hospital-based case–control study, data from 171 participants with UC, 251 participants with CD, and 400 participants with other gastrointestinal conditions (who served as controls) were obtained. All participants were diagnosed in a private polyclinic in Riyadh between January 2009 and December 2017. To be included in the IBD group, participants had to be newly diagnosed with IBD. To be included in the control group, participants had to show no signs of IBD, drug colitis, malignancy, polyposis, or diverticulosis [42,43]. Participants with no data on dairy consumption were excluded, leaving 158 participants with UC, 244 participants with CD, and 395 controls for statistical analysis. All participants were aged ≥18 years.

#### 2.1.2. Assessment of Inflammatory Bowel Disease

As described elsewhere [42,43], participants with IBD manifestations, such as abdominal pain, diarrhea, bloating, loss of appetite, unexplained weight loss, or bloody stool, were subjected to laboratory investigations, including urine and stool analysis for biomarkers of inflammation and serum complete blood count, C-reactive protein, erythrocyte sedimentation rates, bilirubin, alanine aminotransferase, creatinine, and alkaline phosphatase. Participants with manifestations and laboratory findings suggesting IBD underwent gastrointestinal endoscopies. Histopathological analysis of specimens was conducted to confirm the diagnosis.

#### 2.1.3. Assessment of Dairy Consumption

Data on dairy consumption were collected using a self-administered questionnaire distributed before diagnoses were made. The following question was used to assess dairy consumption in a typical week: “How frequently do you consume dairy products?” The available responses were “once per month or less”, “once per week”, “twice per week”, “every day”, “do not remember”, and “NA.” We merged the responses “once per month or less”, “once per week”, and “twice per week” into one category, “infrequent consumption”, to obtain statistical power, while the response “every day” was categorized as “frequent consumption.” Participants with the responses “do not remember” and “NA” were excluded from the analysis. Illiterate participants sought help from the data collectors. Dairy products included milk, yogurt, and cheese. Food frequency questions were not validated or pre-tested.

#### 2.1.4. Statistical Analysis

Logistic regression was used to compute the ORs and 95% confidence intervals (CIs) for IBD, UC, and CD in the case of frequent versus infrequent dairy consumption. The results were adjusted for age, sex, and body mass index (BMI). The results were further stratified by sex. The Statistical Package for Social Science (SPSS) 2013 (IBM SPSS Statistics for Windows, version 22.0, IBM Corporation, Armonk, NY, USA) was used for data analysis.

### 2.2. The Meta-Analysis

#### 2.2.1. Literature Review and Eligibility Criteria

We performed the meta-analysis per the Preferred Reporting Items for Systematic Reviews and Meta-Analysis (PRISMA) guidelines [44]. Two authors independently conducted thorough searches of the Medline (PubMed), Web of Science, and Scopus databases to locate relevant studies published up to 10 June 2024 (Supplementary Table S1). In addition, we manually reviewed the reference lists of the identified studies and related review articles to find any further relevant studies. There were no restrictions on the publication year.

#### 2.2.2. Eligibility Criteria

Studies were included in this meta-analysis if (1) the consumption of dairy or any of its subtypes was the exposure, (2) the outcome was IBD, UC, or CD, (3) participants were adults, and (4) the risk estimate or prevalence of IBD, UC, or CD associated with dairy consumption was provided.

#### 2.2.3. Study Selection

Two authors independently reviewed the titles and abstracts of all studies retrieved from the initial search. These studies were then assessed against the established eligibility criteria to compile the final list for the meta-analysis.

#### 2.2.4. Data Extraction

Two authors independently extracted the following data from the included studies: the last name of the first author, the year of publication, the study location, the sample size, the study design, the types of IBD, the categories of dairy consumption, adjusted variables, and risk estimates. When a given study provided different regression models that adjusted for several variables, we included the model that adjusted for the largest number of variables.

#### 2.2.5. Risk of Bias and Quality Assessment

The quality of the individual studies was evaluated using the modified Newcastle–Ottawa Scale (NOS). Cross-sectional and case–control studies were evaluated based on definitions and representativeness of cases and controls, comparability, and ascertainment of dairy consumption (applying the same method of dairy consumption ascertainment for cases and controls), and nonresponse rate. Cohort studies were evaluated based on the representativeness of the exposed cohort, ascertainment of dairy consumption, selection of the non-exposed cohort (excluding IBD cases at baseline), comparability, IBD assessment, and length of follow-up of outcome [45]. Two authors independently conducted these assessments, resolving any disagreements through discussion and consultation with the other authors.

#### 2.2.6. Statistical Analysis

The random effects model was applied to compute the pooled odds ratio (OR) and 95% confidence interval (95% CI) for IBD, UC, and CD among participants who reported the highest versus the lowest frequencies of dairy consumption [46]. We calculated tau^2^ (estimated amount of total heterogeneity), *I*^2^ (total heterogeneity/total variability), and H^2^ (total variability/sampling variability) statistics to examine statistical heterogeneity across studies [47]. Publication bias was assessed using the regression test for funnel plot asymmetry [48]. We conducted sensitivity analyses by removing studies one by one and combining the remaining studies. The R 3.2.0 statistical package (Metafor: Meta-Analysis Package for R) was used for statistical analysis [49].

## 3. Results

### 3.1. The Case–Control Study

Compared to the control group, participants with UC were older (*p*-value = 0.014), while participants with CD were younger (*p*-value < 0.001). The BMI of participants with UC or CD was lower than that of the controls (*p*-value < 0.001 each) (Table 1). After adjusting for age, sex, and the BMI, frequent dairy consumption was associated with a lower possibility of UC but not CD or IBD: ORs (95% CIs) = 0.64 (0.41, 1.00), 0.97 (0.65, 1.45), and 0.82 (0.58. 1.16), respectively. The association with UC was relatively more obvious among men than women: ORs (95% CIs) = 0.59 (0.33, 1.07) and 0.69 (0.33, 1.46), respectively (Table 2).

### 3.2. The Meta-Analysis

**Figure 1:**
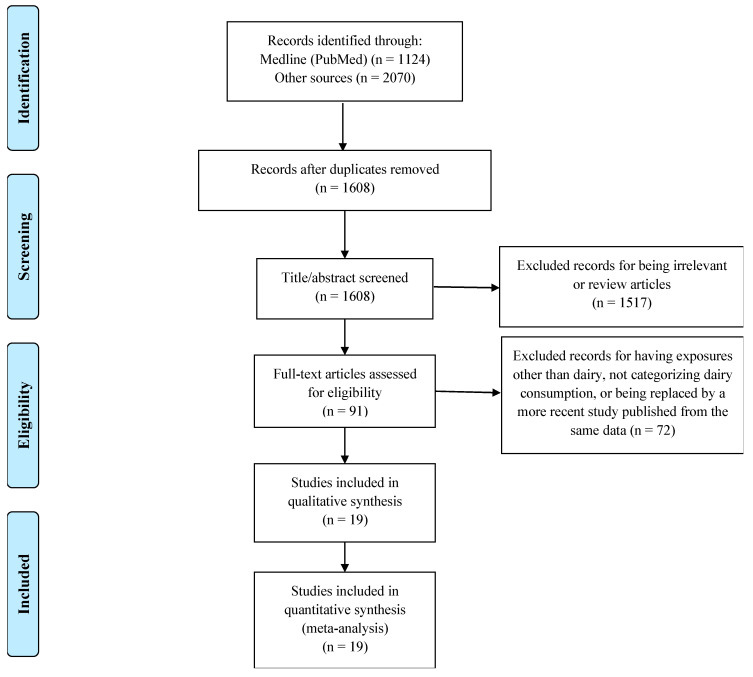
PRISMA flowchart of the studies included in the meta-analysis.

Most studies were excluded for being duplicates or reviews or having irrelevant exposures (dairy consumption) or outcomes (IBD, UC, or CD). Ultimately, we identified 19 studies that met the criteria for inclusion in this meta-analysis (Figure 1). The studies were published between 1991 and 2024. The risk estimates were calculated for dairy consumption as a whole in 11 studies, milk consumption in 7 studies, and cheese consumption in 1 study. The study designs were distributed as follows: 13 case–control studies, 4 prospective cohort studies, and 2 cross-sectional studies. The outcomes investigated were as follows: 15 studies investigated UC, 9 studies investigated CD, and 6 studies investigated IBD as a whole (Table 3). Among the 15 case–control and cross-sectional studies, 3 studies had good quality (scores 7/9), 10 studies had average quality (scores 5–6/9), and 2 studies had poor quality (scores 3/9). However, all cohort studies had good quality, with a minimum risk of major bias (scores 7–9/9) (Table 4).

#### 3.2.1. Ulcerative Colitis

**Figure 2:**
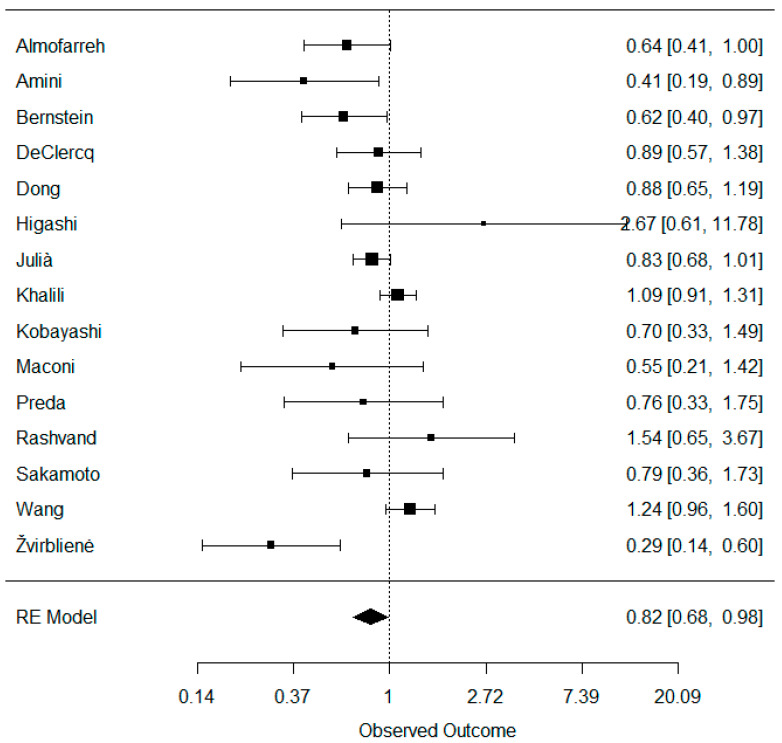
Meta-analysis of the association between dairy consumption and ulcerative colitis.

A total of 15 studies were included in the meta-analysis investigating the association between dairy consumption and UC. Their weights were as follows: Almofarreh 7.9%, Amini 4.1%, Bernstein 8.0%, DeClercq 8.0%, Dong 10.6%, Higashi 1.4%, Julià 12.7%, Khalili 12.9%, Kobayashi 4.2%, Maconi 3.0%, Preda 3.7%, Rashvand 3.4%, Sakamoto 4.0%, Wang 11.6%, and Žvirblienė 4.5%. When combined, dairy consumption was found to be negatively associated with UC: OR (95% CI) = 0.82 (0.68, 0.98) (Figure 2). No publication bias was detected: z = −1.125 and *p* = 0.260 (Supplementary Figure S1). However, a moderate heterogeneity across studies was detected: tau^2^ = 0.059, *I*^2^ = 59.58%, and H^2^ = 2.47. Leaving out studies one by one and combining the remaining studies did not materially affect the heterogeneity. The lowest heterogeneity was noticed when the Žvirblienė study was removed, while the highest heterogeneity was noticed when the DeClercq study was removed: tau^2^ ranged between 0.035 and 0.067, *I*^2^ ranged between 47.62% and 62.46%, and H^2^ ranged between 1.91 and 2.66 (Supplementary Figure S2).

#### 3.2.2. Crohn’s Disease

**Figure 3:**
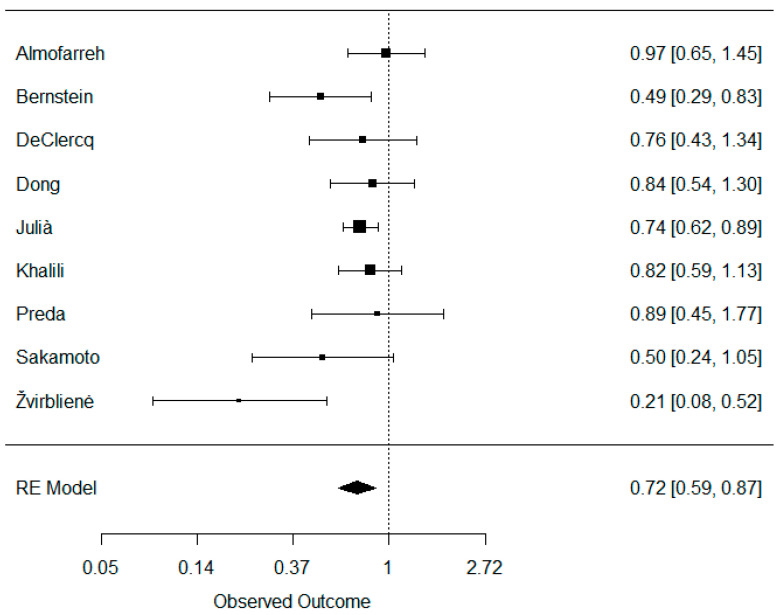
Meta-analysis of the association between dairy consumption and Crohn’s disease.

A total of nine studies were included in the meta-analysis investigating the association between dairy consumption and UC. Their weights were as follows: Almofarreh 13.3%, Bernstein 9.4%, DeClercq 8.5%, Dong 12.0%, Julià 24.3%, Khalili 16.5%, Preda 6.3%, Sakamoto 5.7%, and Žvirblienė 4.0%. When combined, dairy consumption was found to be negatively associated with CD: OR (95% CI) = 0.72 (0.59, 0.87) (Figure 3). No significant signs of publication bias were detected: z = −1.768, *p* = 0.077 (Supplementary Figure S3). However, a moderate heterogeneity across studies was detected: tau^2^ = 0.032, *I*^2^ = 41.16%, and H^2^ = 1.70. Leaving out studies one by one and combining the remaining studies did not significantly affect the heterogeneity. Removing the Žvirblienė study dissolved the heterogeneity across studies: *I*^2^ = 0.0% (Supplementary Figure S4).

#### 3.2.3. Inflammatory Bowel Disease

A total of six studies investigated the association between dairy consumption and IBD as a whole. Their weights were as follows: Almofarreh 24.3%, DeClercq 12.2%, Han 24.3%, Jantchou 13.8%, Mi 2.8%, and Narula 22.5%. When their results were combined, the association between dairy consumption and IBD was statistically non-significant: OR (95% CI) = 0.92 (0.71, 1.17) (Supplementary Figure S5). A moderate heterogeneity across studies was detected: tau^2^ = 0.040, *I*^2^ = 41.41%, and H^2^ = 1.71. However, removing the Narula study strengthened the negative association between dairy consumption and IBD: OR (95% CI) = 0.82 (0.65, 1.03); it also minimized the heterogeneity across studies: tau^2^ = 0.009, *I*^2^ = 11.77%, and H^2^ = 1.13.

## 4. Discussion

The case–control study, investigating Saudi participants, indicated a negative association between dairy consumption and UC only, while the meta-analysis showed a negative association between dairy consumption and both UC and CD. This finding challenges some previous notions that dairy, particularly due to its lactose content, might contribute to the development of IBD in susceptible individuals. Instead, this study supports the hypothesis that dairy products might have protective effects or that the avoidance of dairy might be associated with other dietary patterns that increase IBD risk.

The protective effect of dairy consumption against UC and CD is multi-faceted, involving several interrelated mechanisms. Firstly, dairy products provide probiotic benefits, contributing beneficial bacteria to the gut, which can enhance intestinal health and function [50]. Secondly, the anti-inflammatory properties of specific dairy components, such as casein and whey proteins, can help reduce inflammation in the gut [51]. Additionally, dairy is a rich source of calcium and vitamin D, both of which play crucial roles in maintaining gut integrity and immune function [52,53]. Furthermore, the consumption of dairy leads to the production of short-chain fatty acids (SCFAs) through the fermentation of lactose by gut bacteria. SCFAs, such as butyrate, propionate, and acetate, serve as energy sources for colon cells and have anti-inflammatory effects, contributing to overall gut health [54,55]. Finally, dairy consumption favorably modulates the composition of the gut microbiota, promoting the growth of beneficial bacterial species that can outcompete harmful pathogens and reduce gut inflammation [56]. These diverse mechanisms may work synergistically to maintain gut health and prevent the onset of IBD by reducing inflammation and supporting a balanced gut microbiome.

The finding that dairy consumption is associated with a lower risk of UC and CD has significant clinical implications. Healthcare providers might consider recommending the inclusion of dairy products in the diets of individuals at risk of IBD, provided there are no contraindications, such as lactose intolerance or dairy allergies. Nutritional counseling can emphasize the potential benefits of dairy, including its anti-inflammatory components and essential nutrients, which may promote gut health and modulate immune responses. Preventive strategies could integrate dairy as part of a balanced diet to reduce inflammation and support overall digestive health. Public health policies might incorporate these findings into dietary guidelines, promoting dairy consumption as a preventive measure against IBD, particularly in regions with rising prevalence [57].

Of note, investigating an understudied population, assigning newly diagnosed IBD patients, and using standardized methods for IBD diagnosis are the main strengths of the case–control study. However, it carried several limitations. First, data were collected from patients attending a single polyclinic in Riyadh, the largest urban city in Saudi Arabia. This polyclinic is a private institution, suggesting that our patients might have had a higher socio-economic status. Therefore, caution is needed when generalizing our results to people from rural areas and poorer suburbs. Second, we did not use a validated food frequency questionnaire, which could have affected the accuracy and reliability of dietary intake data. Third, we did not gather information on the various components of dairy, which might have distinct associations with IBD. Dairy consumption was investigated as a whole rather than focusing on specific nutrients, such as vitamin D and calcium, which could have different effects on IBD risk. Fourth, due to the limited number of cases in each dairy consumption frequency group, we combined the groups representing non-daily dairy consumption into one group. As a result, a dose–response association could not be evaluated, limiting our ability to understand how varying levels of dairy intake affect IBD risk. Fifth, while the study used standardized approaches to ascertain IBD diagnosis, IBD activity scores were not calculated. Sixth, the results were not adjusted for several important variables, such as total calorie intake, which could confound the association between dairy consumption and IBD. Seventh, since controls were patients with other gastrointestinal manifestations that could be related to dairy consumption, the association might have been impacted, potentially biasing our findings. Eighth, patients were recruited over a relatively long period, during which popular trends toward dairy consumption might have changed, adding another layer of variability to the data.

Augmenting the number of UC and CD cases by involving a large number of studies was the main strength of the meta-analysis. However, it also had several limitations. First, the included studies varied in design, sample size, and dairy consumption categorization, potentially introducing heterogeneity. Second, the observational nature of these studies prevented the firm establishment of causality. Third, confounding factors, such as overall diet quality, total energy consumption, and lifestyle factors, were not uniformly controlled across studies. Fourth, although the meta-analysis was conducted according to standard procedures, its protocol was not registered in advance.

## 5. Conclusions

Dairy consumption was associated with a decreased risk of UC and CD. Our findings hold implications for dietary recommendations in the context of IBD prevention and management. Health practitioners might consider encouraging the inclusion of dairy products in the diets of individuals at risk of IBD, provided there are no contraindications, such as lactose intolerance or dairy allergies. Future studies should further elucidate the biological mechanisms underlying this association. Investigations could focus on differentiating the impacts of various dairy components, examining the role of the gut microbiome, and exploring genetic and environmental interactions. Additionally, longitudinal studies with larger, more diverse populations and standardized measures of dairy intake would help strengthen the evidence base and refine dietary guidelines for IBD prevention.

## Figures and Tables

**Table 1:** Comparison between cases and controls in the case–control study.

Characteristics	Inflammatory Bowel Disease	Ulcerative Colitis	Crohn’s Disease	Controls
**Overall**				
Number of participants	402	158	244	395
Age (mean ± SD), years	36.5 ± 10.5	39.9 ± 12.6	34.3 ± 8.2	37.7 ± 8.8
Body mass index (mean ± SD), kg/m^2^	23.8 ± 5.5	25.1 ± 5.8	22.9 ± 5.0	27.2 ± 6.4
**Men**				
Number of participants	259	93	166	256
Age (mean ± SD), years	37.1 ± 10.8	40.8 ± 13.5	35.1 ± 8.4	36.3 ± 7.2
Body mass index (mean ± SD), kg/m^2^	23.4 ± 5.1	24.3 ± 5.0	22.9 ± 5.1	27.0 ± 6.5
**Women**				
Number of participants	143	65	78	139
Age (mean ± SD), years	35.3 ± 9.8	38.5 ± 11.2	32.7 ± 7.5	40.1 ± 10.8
Body mass index (mean ± SD), kg/m^2^	24.4 ± 5.9	26.3 ± 6.6	22.9 ± 4.8	27.5 ± 6.3

**Table 2:** Association between dairy consumption and inflammatory bowel disease in the case–control study.

	Frequent Dairy Consumption	Infrequent Dairy Consumption	Adjusted OR (95% CI)
**Overall**	n = 588 (%)	n = 209 (%)	--
Control	301 (51.2)	94 (45.0)	1 (Reference)
Inflammatory bowel disease	287 (48.8)	115 (55.0)	0.82 (0.58, 1.16)
Ulcerative colitis	114 (19.4)	44 (21.0)	0.64 (0.41, 1.00)
Crohn’s disease	173 (29.4)	71 (34.0)	0.97 (0.65, 1.45)
**Men**	n = 378 (%)	n = 137 (%)	--
Control	189 (50.0)	67 (48.9)	1 (Reference)
Inflammatory bowel disease	189 (50.0)	70 (51.1)	0.81 (0.53, 1.25)
Ulcerative colitis	67 (17.7)	26 (19.0)	0.59 (0.33, 1.07)
Crohn’s disease	122 (32.3)	44 (32.1)	0.99 (0.61, 1.61)
**Women**	n = 210 (%)	n = 72 (%)	--
Control	112 (53.3)	27 (37.5)	1 (Reference)
Inflammatory bowel disease	98 (46.7)	45 (62.5)	0.79 (0.43, 1.44)
Ulcerative colitis	47 (22.4)	18 (25.0)	0.69 (0.33, 1.46)
Crohn’s disease	51 (24.3)	27 (37.5)	0.91 (0.45, 1.85)

ORs (95% CIs) were adjusted for age, sex, and the body mass index.

**Table 3:** Summary of the studies included in the meta-analysis.

Study ID	Study Design	Population	Dairy Consumption Assessment and Categories	Covariates
Almofarreh (2024) Saudi Arabia	Case–control	158 UC cases (39.9 ± 12.6 years), 244 CD cases (34.3 ± 8.2 years), and 395 controls (37.7 ± 8.8 years) from a gastroenterology clinic in Riyadh	Self-administered questionnaire Dairy consumption (milk, yogurt, and cheese) every day vs. less frequently	Age, sex, and BMI
Amini (2024) Iran [11]	Case–control	340 UC cases (36.5 ± 11.7 years, 38.8% men) and 782 controls (43.7 ± 14.5 years, 40.2% men) from 3 gastroenterology clinics in Tehran	Food frequency questionnaire Dairy consumption > 395 vs. < 118 g/day	Age; energy; sex; BMI; physical activity; alcohol; smoking; and intake of meat, grains, legumes, nuts, fruits, and vegetables
Bernstein (2006) Canada [12]	Case–control	217 UC cases and 364 CD cases, aged 18–50 years, from the University of Manitoba IBD Research Registry and 433 controls from the Manitoba Health Registry, matched by age, gender, and residence	Questionnaire Drinking unpasteurized milk as a child (high vs. low)	Age and sex
DeClercq (2018) Canada [13]	Cross sectional	12,462 without IBD, 111 CD cases, and 119 UC cases from the Atlantic Partnership for Tomorrow’s Health (PATH) study, aged 30–74 years	Food frequency questionnaire Meeting milk and dairy consumption guidelines	None
Dong (2022) 8 European countries [14]	Prospective cohort	Among 413,593 participants from the EPIC cohort, 177 incident CD cases and 418 incident UC cases identified within a mean follow-up duration of 16.8 years	Food frequency questionnaire Dairy consumption > 462 vs. < 184 g/day	Center, age, sex, BMI, smoking, physical activity, energy, and education
Han (2015) US [15]	Cross sectional	33,672 adults from the 2015 National Health Interview Survey (NHIS), aged 18–85 years, with 44.8% men and 1.3% with IBD	Diet and Nutrition Questionnaire from the Cancer Control Module (CCM) Milk consumption/month > vs. ≤ median values	Age, race, sex, ethnicity, poverty, region, alcohol, smoking, and BMI
Higashi (1991) Japan [16]	Case–control	50 UC cases from 3 hospitals in Japan, aged ≥18 years, and 50 controls matched by age and sex	Interview (food consumption during the pre-illness year) Dairy consumption ≥ 3 times/week vs. less frequently	None
Jantchou (2010) France [17]	Prospective cohort	Among 67,581 women, aged 40–65 years, from the E3N cohort, 30 incident CD cases and 43 incident UC cases identified, within a mean follow-up duration of 10.4 years	Diet history questionnaire Highest (mean = 511.4 g/day) vs. lowest categories of dairy consumption (mean = 131.3 g/day)	Energy and intake of meat, fish, and eggs
Julià (2021) Spain [18]	Case–control	1968 healthy controls (49.6 ± 7.0 years, 59% men), 1407 UC cases (48.7 ± 14.5 years, 55% men), and 1946 CD cases (41.9 ± 7.0 years, 50% men) from the Immune-Mediated Inflammatory Diseases Consortium (IMIDC)	Food frequency questionnaire Dairy consumption 6–7 times/week vs. less frequently	Age, sex, smoking, region, season, education, and physical activity
Khalili (2020) Sweden [19]	Prospective cohort	Among 83,147 participants, aged 45–79 years, from the Cohort of Swedish Men and Swedish Mammography Cohort, 164 incident CD cases and 395 incident UC cases identified, with an average follow-up of 17 years	Food frequency questionnaire Above vs. below median consumption of fermented dairy	Sex, age, education, BMI, smoking, and total caloric intake
Kobayashi (2020) Japan [20]	Case–control	83 UC cases (42.0 ± 14.4 years, 55% men) and 128 age- and sex-matched control patients from 38 hospitals	Diet history questionnaire Dairy consumption ≥ 78.8 vs. < 26.2 g/4184 kJ/day	BMI, smoking, alcohol, family history of UC, and appendicitis history
Maconi (2010) Italy [21]	Case–control	83 UC cases (37.5 ± 15.2 years, 59% men) from the gastroenterology department of a university hospital and 160 controls (40.0 ± 14.6 years, 60.6% men)	Questionnaire Highest vs. lowest tertile of milk consumption	Age, sex, education, smoking, and BMI
Mi (2022) China [22]	Case–control	50 IBD cases from a tertiary referral center and 50 controls matched for age and sex	Food frequency questionnaire Milk consumption 3 times/week vs. not at all or occasionally	Intake of chili, fish, nuts, eggs, and fruits
Narula (2021) 21 countries [23]	Prospective cohort	116,087 adults, aged 35–70 years, from the Prospective Urban Rural Epidemiology (PURE) cohort	Food frequency questionnaire Dairy consumption > 2 vs. < 1 serving/day	Age, sex, region, education, alcohol, smoking, BMI, energy, and location
Preda (2023) Romania [24]	Case–control	89 CD cases (42 years, 38.2% men), 40 UC cases (41.5 years, 55% men), and 64 controls (45.5 years, 42.2% men)	Dietary questionnaire Consuming cheese every day vs. less frequently	None
Rashvand (2015) Iran [25]	Case–control	62 UC cases (37.4 years, 44% men) and 124 controls (36.2 years, 44% men)	Food frequency questionnaire Highest vs. lowest tertiles of dairy consumption	Energy; H. pylori infection; appendectomy history; and intake of fat, carbohydrate, and food groups
Sakamoto (2005) Japan [26]	Case–control	108 UC cases (51.9% men), 126 CD cases (72.2%), and 211 controls (64% men) matched for sex, age, and hospital, aged 15–34 years	Food frequency questionnaire Dairy consumption > 259.8 vs. < 54.3 g/day	Age, sex, area, energy, education, and smoking
Wang (2013) China [27]	Case–control	1308 UC cases from 17 hospitals in 12 areas and 1308 age- and sex-matched controls	Questionnaire Milk consumption (heavy vs. rare)	None
Žvirblienė (2006) Lithuania [28]	Case–control	101 UC (56 men (44 years) and 45 women (43 years)), 44 CD (21 men (46 years) and 23 women (44 years)), and 178 healthy controls (86 men (41 years) and 92 women (44 years))	Self-report questionnaire Daily milk consumption vs. less frequently	None

**Table 4:** Quality assessment of the studies included in the meta-analysis using the Newcastle–Ottawa Scale.

Cross-Sectional and Case–Control Studies								
Item	Almofarreh	Amini	Bernstein	DeClercq	Han	Higashi	Julià	Kobayashi
Case definition	*	*	*	--	--	*	*	*
Representativeness of cases	*	*	*	--	--	*	*	*
Selection of controls	--	*	*	*	*	*	*	--
Definition of controls	*	*	*	*	*	*	*	*
Comparability	*	*	*	--	*	*	*	*
Ascertainment of exposure	--	*	--	--	*	--	*	*
The same method of ascertainment for cases and controls	*	*	*	*	*	*	*	*
Nonresponse rate	--	--	--	--	--	--	--	--
Overall (total number of asterisks)	5	7	6	3	5	6	7	6
Item	Maconi	Mi	Preda	Rashvand	Sakamoto	Wang	Žvirblienė	
Case definition	*	*	--	--	*	*	*	
Representativeness of cases	*	*	*	*	*	*	*	
Selection of controls	*	--	--	--	--	*	*	
Definition of controls	*	*	*	*	*	*	*	
Comparability	*	*	--	*	**	*	--	
Ascertainment of exposure	*	*	--	*	*	--	--	
The same method of ascertainment for cases and controls	*	*	*	*	*	*	*	
Nonresponse rate	--	--	--	--	--	--	--	
Overall (total number of asterisks)	7	6	3	5	6	6	5	
Cohort studies								
Item	Dong	Jantchou	Khalili	Narula				
Representativeness of the exposed cohort	*	*	*	*				
Ascertainment of exposure	*	*	*	*				
Selection of the non-exposed cohort	*	*	*	*				
Demonstration that the outcome of interest was not present at the start of the study	*	*	*	*				
Comparability	**	--	**	**				
Assessment of outcome	*	*	*	--				
Follow-up long enough for outcomes to occur	*	*	*	*				
Adequacy of follow-up of cohorts	*	*	*	*				
Overall (total number of asterisks)	9	7	9	8				

The possible overall scores range between 0 and 9. Every * represents 1 point.

## Data Availability

Upon reasonable request, data will be provided by the corresponding author after consulting the institutional review board of King Fahd Medical City.

## Supplementary Materials

The following supporting information can be downloaded at: https://www.mdpi.com/article/10.3390/nu16162747/s1. Table S1. PubMed search strategy. Figure S1. Funnel plot of the studies investigating the association between dairy consumption and ulcerative colitis. Figure S2. The impact of removing studies one by one and combining the remaining studies on the meta-analysis investigating the association between dairy consumption and ulcerative colitis. Figure S3. Funnel plot of the studies investigating the association between dairy consumption and Crohn’s disease. Figure S4. The impact of removing studies one by one and combining the remaining studies on the meta-analysis investigating the association between dairy consumption and Crohn’s disease. Figure S5. Meta-analysis of the association between dairy consumption and inflammatory bowel disease.
