# Supplementary material for: Dairy Consumption and Inflammatory Bowel Disease among Arab Adults: A Case–Control Study and Meta-Analysis
Source: Nutrients. 2024 Aug 17;16(16):2747. doi: 10.3390/nu16162747 (PMC11357007; doi:10.3390/nu16162747)
Supplement: Supplementary file 1 [file nutrients-16-02747-s001.zip › nutrients-3149675-supplementary.pdf]

nutrients MDPI

Table S1. PubMed search strategy.

Search: (Milk OR Dairy) AND (Inflammatory bowel disease OR Ulcerative colitis OR Crohn's disease)<br>("milk, human"[MeSH Terms] OR ("milk"[All Fields] AND "human"[All Fields]) OR "human milk"[All Fields] OR "milk"[All Fields] OR "milk"[MeSH Terms] OR ("dairies"[All Fields] OR "dairy"[All Fields] OR "dairy s"[All Fields] OR "dairying"[MeSH Terms] OR "dairying"[All Fields])) AND ("inflammatory bowel diseases"[MeSH Terms] OR ("inflammatory"[All Fields] AND "bowel"[All Fields] AND "diseases"[All Fields]) OR "inflammatory bowel diseases"[All Fields] OR ("inflammatory"[All Fields] AND "bowel"[All Fields] AND "disease"[All Fields]) OR "inflammatory bowel disease"[All Fields] OR ("colitis, ulcerative"[MeSH Terms] OR ("colitis"[All Fields] AND "ulcerative"[All Fields]) OR "ulcerative colitis"[All Fields] OR ("ulcerative"[All Fields] AND "colitis"[All Fields])) OR ("crohn disease"[MeSH Terms] OR ("crohn"[All Fields] AND "disease"[All Fields]) OR "crohn disease"[All Fields] OR "crohn s disease"[All Fields]))
Translations<br>Milk: "milk, human"[MeSH Terms] OR ("milk"[All Fields] AND "human"[All Fields]) OR "human milk"[All Fields] OR "milk"[All Fields] OR "milk"[MeSH Terms]<br>Dairy: "dairies"[All Fields] OR "dairy"[All Fields] OR "dairy's"[All Fields] OR "dairying"[MeSH Terms] OR "dairying"[All Fields]<br>Inflammatory bowel disease: "inflammatory bowel diseases"[MeSH Terms] OR ("inflammatory"[All Fields] AND "bowel"[All Fields] AND "diseases"[All Fields]) OR "inflammatory bowel diseases"[All Fields] OR ("inflammatory"[All Fields] AND "bowel"[All Fields] AND "disease"[All Fields]) OR "inflammatory bowel disease"[All Fields]<br>Ulcerative colitis: "colitis, ulcerative"[MeSH Terms] OR ("colitis"[All Fields] AND "ulcerative"[All Fields]) OR "ulcerative colitis"[All Fields] OR ("ulcerative"[All Fields] AND "colitis"[All Fields])<br>Crohn's disease: "crohn disease"[MeSH Terms] OR ("crohn"[All Fields] AND "disease"[All Fields]) OR "crohn disease"[All Fields] OR "crohn s disease"[All Fields]

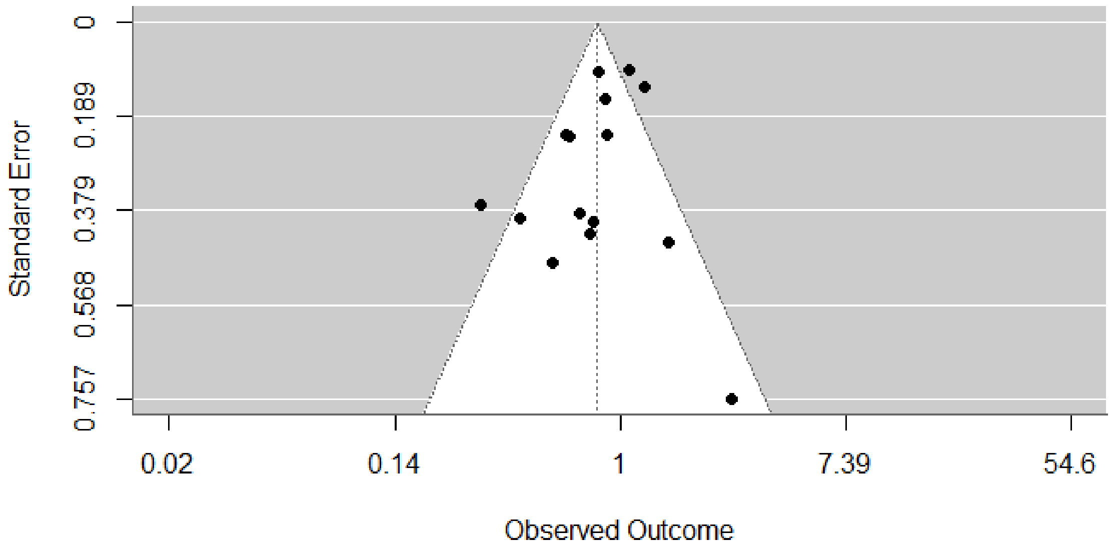

Figure S1. Funnel plot of the studies investigating the association between dairy consumption and ulcerative colitis.

	estimate	se	zval	pval	ci.lb	ci.ub	Q	Qp	tau2	I2	H2
Almofarreh	-0.179	0.098	-1.838	0.066	-0.371	0.012	32.238	0.002	0.059	59.675	2.480
Amini	-0.167	0.092	-1.814	0.070	-0.347	0.013	30.517	0.004	0.052	57.401	2.347
Bernstein	-0.175	0.097	-1.809	0.071	-0.365	0.015	31.674	0.003	0.057	58.957	2.436
DeClercq	-0.212	0.101	-2.098	0.036	-0.411	-0.014	34.629	0.001	0.067	62.459	2.664
Dong	-0.216	0.105	-2.065	0.039	-0.422	-0.011	34.595	0.001	0.072	62.422	2.661
Higashi	-0.215	0.093	-2.307	0.021	-0.398	-0.032	32.592	0.002	0.057	60.112	2.507
Julià	-0.215	0.110	-1.958	0.050	-0.430	0.000	33.573	0.001	0.081	61.279	2.583
Khalili	-0.246	0.103	-2.377	0.017	-0.449	-0.043	29.488	0.006	0.066	55.914	2.268
Kobayashi	-0.195	0.097	-2.007	0.045	-0.386	-0.005	34.177	0.001	0.062	61.963	2.629
Maconi	-0.189	0.096	-1.973	0.048	-0.376	-0.001	33.559	0.001	0.060	61.263	2.581
Preda	-0.200	0.097	-2.054	0.040	-0.391	-0.009	34.461	0.001	0.063	62.276	2.651
Rashvand	-0.223	0.095	-2.337	0.019	-0.410	-0.036	33.189	0.002	0.059	60.830	2.553
Sakamoto	-0.201	0.098	-2.064	0.039	-0.393	-0.010	34.516	0.001	0.063	62.337	2.655
Wang	-0.251	0.096	-2.611	0.009	-0.439	-0.063	27.706	0.010	0.052	53.079	2.131
Žvirblienė	-0.137	0.083	-1.659	0.097	-0.299	0.025	24.818	0.024	0.035	47.620	1.909

Figure S2. The impact of removing studies one by one and combining the remaining studies on the meta-analysis investigating the association between dairy consumption and ulcerative colitis.

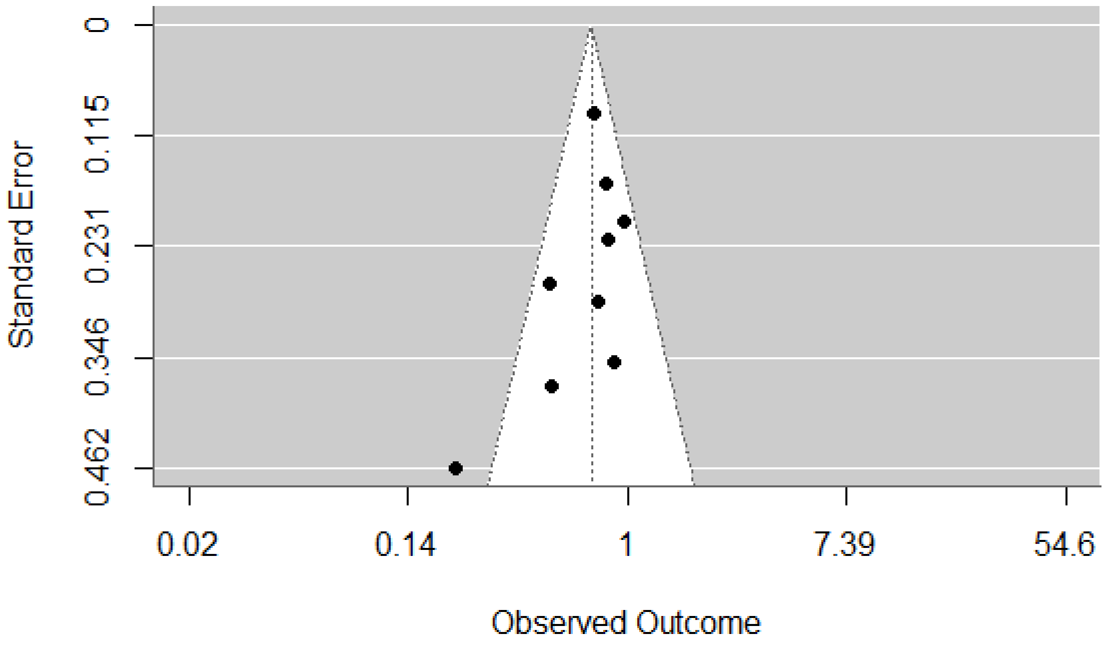

Figure S3. Funnel plot of the studies investigating the association between dairy consumption and Crohn’s disease.

	estimate	se	zval	pval	ci.lb	ci.ub	Q	Qp	tau2	I2	H2
Almofarreh	-0.376	0.106	-3.537	0.000	-0.585	-0.168	11.670	0.112	0.032	40.018	1.667
Bernstein	-0.287	0.099	-2.891	0.004	-0.481	-0.092	11.111	0.134	0.026	37.001	1.587
DeClercq	-0.343	0.110	-3.107	0.002	-0.560	-0.127	13.588	0.059	0.041	48.485	1.941
Dong	-0.360	0.113	-3.194	0.001	-0.580	-0.139	13.253	0.066	0.041	47.182	1.893
Julià	-0.362	0.132	-2.742	0.006	-0.621	-0.103	13.597	0.059	0.064	48.516	1.942
Khalili	-0.367	0.118	-3.101	0.002	-0.600	-0.135	13.153	0.068	0.046	46.781	1.879
Preda	-0.351	0.107	-3.273	0.001	-0.560	-0.141	13.313	0.065	0.038	47.419	1.902
Sakamoto	-0.309	0.103	-3.008	0.003	-0.511	-0.108	12.475	0.086	0.033	43.890	1.782
Žvirblienė	-0.276	0.065	-4.257	0.000	-0.402	-0.149	6.001	0.540	0.000	0.000	1.000

Figure S4. The impact of removing studies one by one and combining the remaining studies on the meta-analysis investigating the association between dairy consumption and Crohn’s disease.

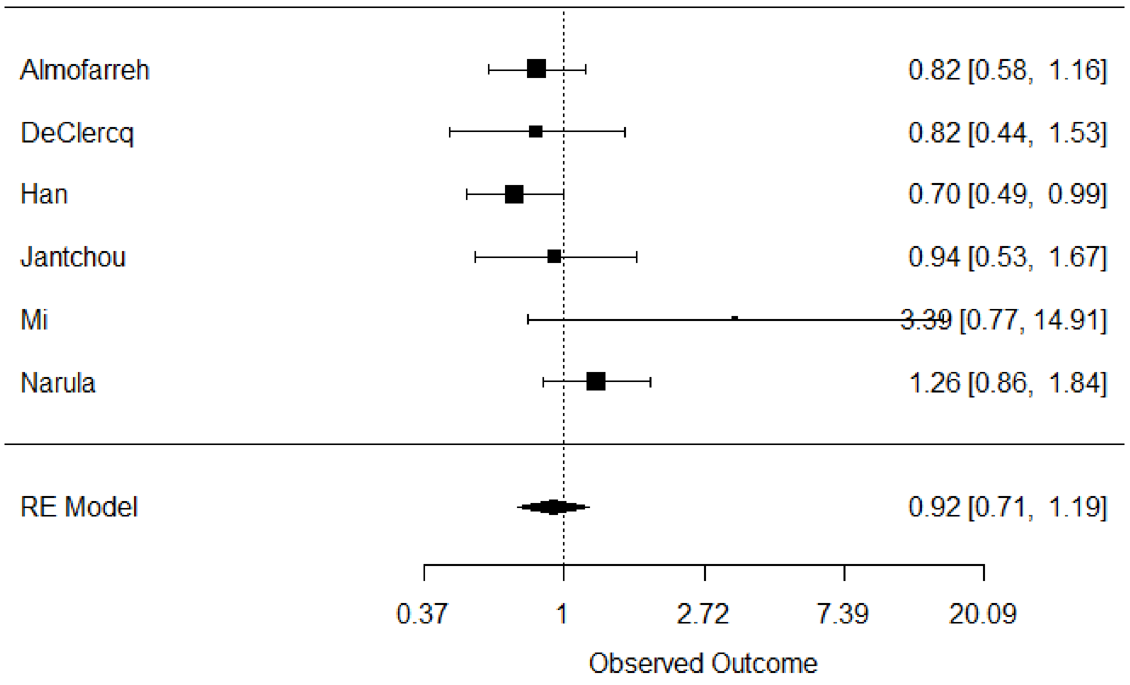

Figure S5. Meta-analysis of the association between dairy consumption and inflammatory bowel disease.
